# Comparison of virulence of *Francisella tularensis* ssp. *holarctica* genotypes B.12 and B.FTNF002-00

**DOI:** 10.1186/s12917-017-0968-9

**Published:** 2017-02-10

**Authors:** Zsuzsa Kreizinger, Károly Erdélyi, Orsolya Felde, Massimo Fabbi, Kinga M. Sulyok, Tibor Magyar, Miklós Gyuranecz

**Affiliations:** 1 Institute for Veterinary Medical Research, Centre for Agricultural Research, Hungarian Academy of Sciences, Hungária körút 21, H-1143 Budapest, Hungary; 2 Veterinary Diagnostic Directorate, National Food Chain Safety Office, P.O. Box 21581, Budapest, Hungary; 3 Istituto Zooprofilattico Sperimentale della Lombardia e dell’Emilia Romagna Bruno Ubertini, National Reference Laboratory for Tularemia, Pavia, 27100 Italy

**Keywords:** Experimental infection, B.FTNF002-00, B.12, B.13, Brown hare, Fischer 344 rat, Tularemia

## Abstract

**Background:**

Two main genetic groups (B.12 and B.FTNF002-00) of *Francisella tularensis* ssp. *holarctica* are endemic in Europe. The B.FTNF002-00 group proved to be dominant in Western European countries, while strains of the B.12 group were isolated mainly in Northern, Central and Eastern Europe. The clinical course of tularemia in the European brown hare (*Lepus europaeus*) also shows distinct patterns according to the geographical area. Acute course of the disease is observed in hares in Western European countries, while signs of sub-acute or chronic infection are more frequently detected in the eastern part of the continent. The aim of the present study was to examine whether there is any difference in the virulence of the strains belonging to the B.FTNF002-00 and B.12 genetic clades.

**Results:**

Experimental infection of Fischer 344 rats was performed by intra-peritoneal injection of three dilutions of a Hungarian (B.12 genotype) and an Italian (B.FTNF002-00 genotype) *F. tularensis* ssp. *holarctica* strain. Moderate difference was observed in the virulence of the two genotypes. Significant differences were observed in total weight loss values and scores of clinical signs between the two genotypes with more rats succumbing to tularemia in groups infected with the B.FTNF002-00 genotype.

**Conclusions:**

Results of the experimental infection are consistent with previous clinical observations and pathological studies suggesting that *F. tularensis* ssp. *holarctica* genotype B.FTNF002-00 has higher pathogenic potential than the B.12 genotype.

**Electronic supplementary material:**

The online version of this article (doi:10.1186/s12917-017-0968-9) contains supplementary material, which is available to authorized users.

## Background

*Francisella tularensis*, the aetiological agent of tularemia is a Gram-negative, zoonotic bacterium with a broad host spectrum showing preference for infecting Lagomorphs and Rodents. *F. tularensis* ssp. *tularensis* is endemic in North America and it has two subpopulations (A.I -containing three major subgroups: A.I.3, A.I.8 and A.I.12- and A.II), which differ in their geographic distribution, host and vector preference, virulence and genetic characteristics [1, 2]. *F. tularensis* ssp. *holarctica* is moderately virulent; it is endemic all over the Northern Hemisphere, but despite its wide distribution, isolates within this subspecies show little genetic diversity [1]. High resolution molecular techniques (whole genome sequencing with single-nucleotide polymorphism (SNP) detection) differentiated two main genetic groups of *F. tularensis* ssp. *holarctica* in Europe (B.12 and B.FTNF002-00) with distinct geographic distribution [1]. The B.FTNF002-00 group proved to be dominant in Western European countries (France, Italy, the Netherlands, Spain), and the B.12 group was isolated mainly in Northern, Central and Eastern Europe (Austria, Czech Republic, Finland, Hungary, Norway, Romania, Slovakia, Sweden, Ukraine) and the European part of Russia, while both genotypes were detected in Germany and Switzerland [1, 3–11]. Whole genome analysis of B.FTNF002-00 and B.12 genotypes revealed distinct genetic differences (e.g. smaller overall genome size of genotype B.FTNF002-00 compared to B.12; differences in gene sizes or orientations and the effects of SNPs in protein coding) which may be the result of a selection process acting on B.FTNF002-00 potentially leading to enhanced virulence and replication potential of this genotype [12]. Differences can be detected in pathological signs of tularemia in European brown hares (*Lepus europaeus*, the reservoir species of the bacterium in Central Europe) in distinct geographical regions in Europe. In Hungary (Eastern Europe), necropsy findings in hares generally include granulomatous lesions in the lung, pericardium and kidneys due to a sub-acute or chronic infection [13], while signs of an acute clinical course (splenomegaly, congestion and haemorrhagic lesions of organs) have been described in hares that succumbed to tularemia in France and in the Netherlands [14, 15]. Results of a recent publication confirmed that lesions observed in brown hares infected by *F. tularensis* ssp. *holarctica* genotype B.12 differ from those associated with the genotype B.FTNF002-00 in Switzerland where both genotypes are present in nature [16].

The use of Fischer 344 rats was preferred in previous experimental *Francisella* infections when feasibility of vaccination or the host’s early innate immune response were examined. Fischer 344 rats reflected best the human susceptibility to tularemia and showed higher resistance to the pathogen compared to other laboratory animals (e.g. mice) [17–20]. Difference in susceptibility of Fischer 344 rats to *F. tularensis* ssp. *tularensis* wild and reference (SCHU S4) strains, and *F. tularensis* ssp. *holarctica* wild (strain from the B.12 clade) and live vaccine (LVS) strains was also described earlier [18]. As intra-peritoneal challenge was shown to be the most successful route in experimental infections with *Francisella* spp., it is considered to be an appropriate way to highlight differences in the hosts’ susceptibility to tularemia or in the virulence of bacterial strains [18, 21].

In this study the virulence of B.FTNF002-00 and B.12 *F. tularensis* ssp. *holarctica* genotypes was compared in Fischer 344 rats performing experimental infection by the intra-peritoneal route.

## Methods

### Bacterial strains

Genotype B.FTNF002-00 *F. tularensis* ssp. *holarctica* strain was originally isolated in 2006 from a European brown hare in Italy (strain ID: PV/21851/2006). Genotype B.12 was isolated in 2008 from a European brown hare in Hungary (strain ID: FTH24/08). Strain PV/21851/2006 was subcultured three times before the experiments, while strain FTH24/08 was gained after a mouse passage and three subcultures. Bacterial strains were cultured on modified Francis agar (chocolate agar supplemented with 1% glucose and 0.1% cysteine) for 48 h at 37 °C and 5% CO_2_. Colonies from these cultures were first suspended in sterile saline and adjusted to 0.5 McFarland turbidity. Subsequently 100 μl of each dilution from a tenfold dilution series of the previous suspension was inoculated on modified Francis agar and incubated for 48 h to determine the number of colony forming units (CFU; 4 × 10^7^). For the infection of the Fischer 344 rats, fresh bacterial colonies were suspended in sterile saline and adjusted to 0.5 McFarland turbidity, and then diluted in sterile saline to obtain 10^0^, 10^1^ and 10^2^ CFU suspensions. Following the experimental infection of the rats, CFUs were checked from the used dilutions on modified Francis plates after 48 h of incubation.

### Experimental infection

Age matched (7 weeks) female Fischer 344 rats (Charles River Laboratories Inc., Wilmington, MA, USA) were used in the study. The animals were kept in accordance with all national and institutional regulations. The animal experiment was performed according to the guidelines approved by the National Ethical Committee (permit number: PEI/001/1927-4/2015) and the ethical committee of the Institute for Veterinary Medical Research. The rats (6 animals/group) were injected via the intra-peritoneal route with 100 μl inocula containing either 10^0^, 10^1^ and 10^2^ CFUs of the B.FTNF002-00 or B.12 genotypes of *F. tularensis* ssp. *holarctica*. A group of 6 Fischer 344 rats injected intra-peritoneally with 100 μl sterile saline was used as a negative control in the experiment. After infection, the animals were checked and measured at 24 h intervals by the same person for 21 days. Clinical signs were registered throughout the experiment and they were used to establish three categories (mild, moderate and severe) of disease severity to be used in the analyses (Table 1). Rats that did not succumb to the infection were euthanized by CO_2_ overexposure at the end of the experiment. Slide agglutination test was performed at necropsy with heart blood, using the commercially available *F. tularensis* antigen (Bioveta, a.s., Ivanovicena Hané, Czech Republic). After a gross pathological examination, tissue samples were collected from the lungs, thymus, liver, spleen, kidneys, small and large intestine, muscle, bone marrow and brain (cerebrum and cerebellum), fixed in 8% neutral buffered formalin and submitted for histological and immunohistochemical examinations.

**Table 1:** Categories of clinical signs shown by *Francisella tularensis* ssp. *holarctica* infected Fischer 344 rats

Severity of disease	Clinical signs
Mild	weight loss, accumulation of porphyrin around the eyes (one or both sides), nasal discharge
Moderate	weight loss, marked porphyrin secretion around the eyes (one or both sides), nasal discharge, ruffled fur, decreased activity, diarrhoea
Severe	weight loss, marked porphyrin secretion around the eyes (both sides), nasal discharge, ruffled fur, inactivity, diarrhoea, laboured breathing, weakness

### Histology and immunohistochemistry

Pathological changes were studied by light microscopy on four-micrometer thick sections of formalin-fixed, paraffin-embedded tissue samples stained with haematoxylin and eosin. Immunohistochemical examinations were performed as described before [22]. In brief, sections were de-paraffinized and incubated in 3% H_2_O_2_ solution for 10 min and in 2% solution of skimmed milk powder for 20 min. The sections were incubated overnight at 4 °C with anti-*F. tularensis* hyperimmune rabbit serum diluted 1:30,000 in phosphate-buffered saline (PBS). Antibody binding was detected using anti-rabbit antibodies with horse-radish peroxidase (HRP)-labelled polymer following manufacturer’s instructions (EnVisionTM + Kit; Dako, Denmark). Non-infected rat tissue samples served as a negative control. For some sections antiserum was replaced by PBS to rule out the possibility of any nonspecific binding. Lesions and immunoreactivity were evaluated by a single pathologist in a treatment-blinded manner. The amount of antigen in the lung, liver and spleen was graded based on the number of affected organs and antigen observations/field of vision at 100x magnification (scored as minimal: <10 observations in only one organ; low: <10 observations in two or three organs; moderate: 10–20 observations in two or three organs; high: >20 observations in all three organs).

### Statistical analysis

The results of the experimental infection experiments were compared with independent *t*-test, Mann-Whitney *U* test and Kruskal-Wallis test in Statistica software (Dell Software, Version 13.1., Aliso Viejo: Dell Inc., CA, USA). The virulence of the B.FTNF002-00 and B.12 groups was compared based on the statistical analyses of the total weight loss and severity of clinical signs (graded as none = 0, mild = 1, moderate = 2 and severe = 3). Total weight loss represents the percent change in body weight on the day when the smallest weight was measured (minimum weight) relative to the weight of the day of infection (initial weight) for each animal (Additional file 1). Clinical sign scores recorded between days 1 and 13 post infection were compared between groups infected with the two genotypes using Mann-Whitney *U* test (Additional file 1).

## Results

**Fig. 1:**
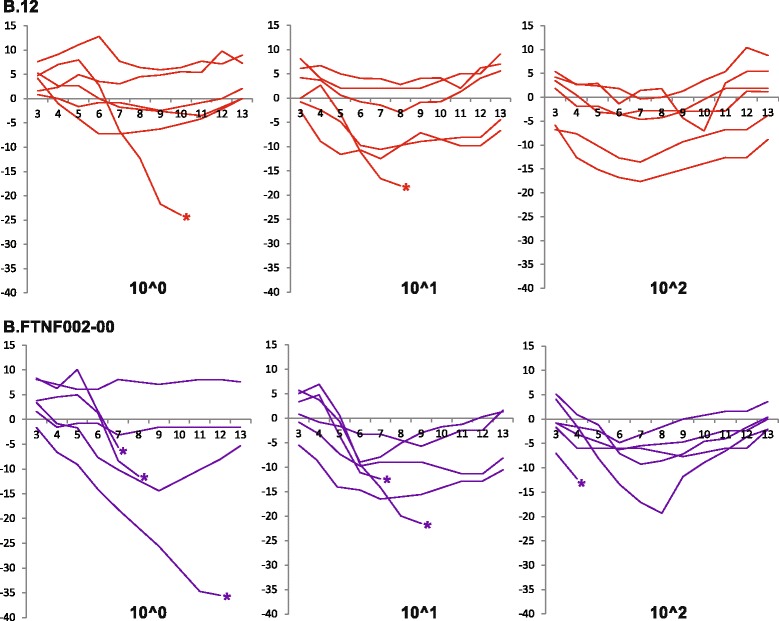
Comparison of changes in body weight in *Francisella tularensis* ssp. *holarctica* infected Fischer 344 rats. Fischer 344 rats were infected with three concentrations of *F. tularensis* ssp. *holarctica* genotype B.12 (*red*) or B.FTNF002-00 (*purple*). Challenge doses are given under each diagram in colony forming units. Values are representing the percent change in body weight between days 3 and 13 post infection relative to the initial weight on the day of infection for each animal. Positive values representing weight gain, while negative values are showing weight loss. Asterisks are representing the death of the animals

**Fig. 2:**
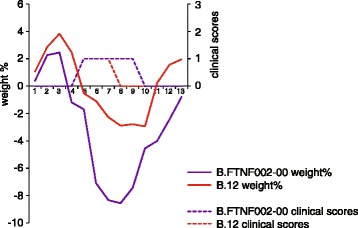
Median values of changes in body weight and clinical sign scores in experimentally infected rats. Fischer 344 rats were infected with *Francisella tularensis* ssp. *holarctica* genotype B.12 or B.FTNF002-00. Values are representing the median of the clinical sign scores (*dashed lines*) and the median percentages of body weight changes (*solid lines*) between days 1 and 13 post infection related to the groups infected with B.12 (*red*) or B.FTNF002-00 (*purple*) genotypes

**Fig. 3:**
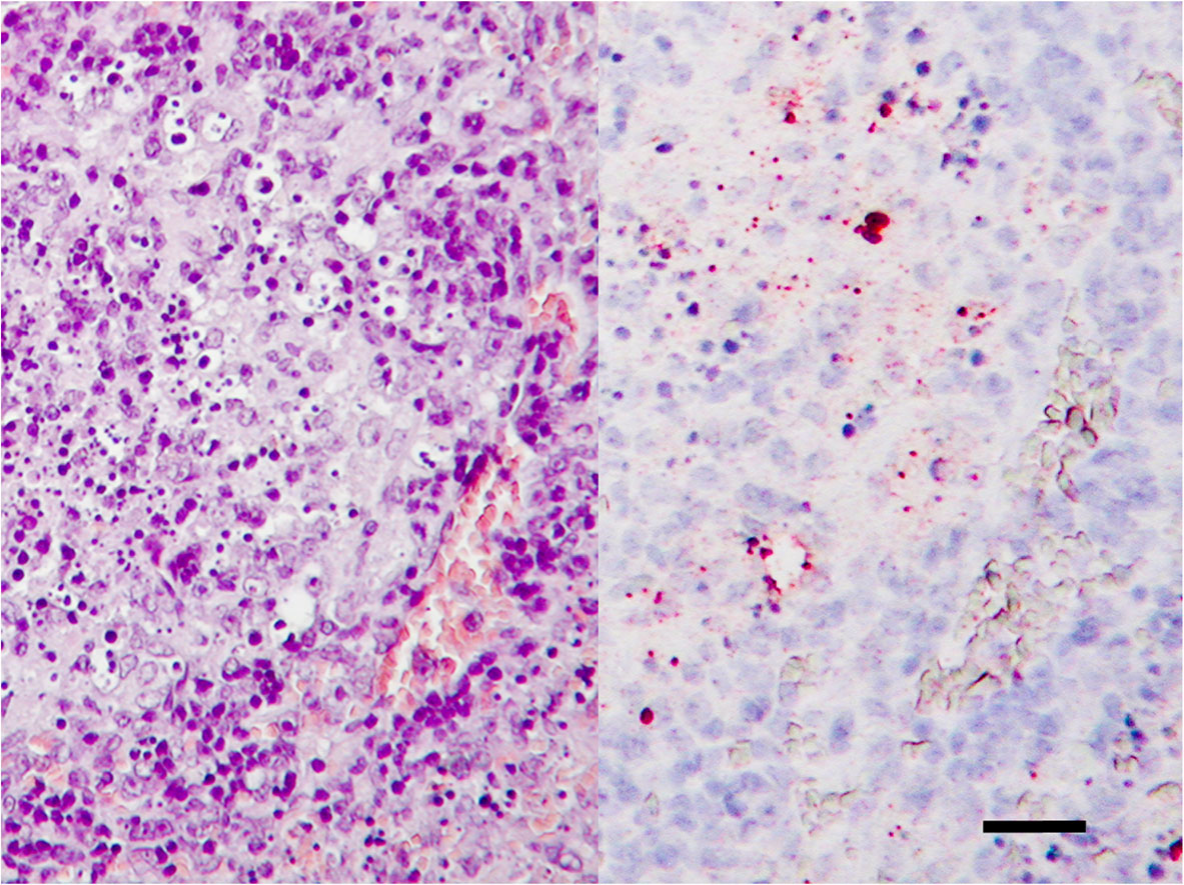
Histological picture of the spleen of a *Francisella tularensis* ssp. *holarctica* genotype B.FTNF002-00 infected Fischer 344 rat. Typical necrotic foci (hematoxilin-eosin, *left*) and the accumulation of *F. tularensis* antigen primarily at the edges of the necrotic area (immunohistochemistry, *right*). Bar = 50 μm

All rats showed clinical signs between days 3–12 post infection after intra-peritoneal inoculation with suspensions containing either 10^0^, 10^1^ and 10^2^ CFUs of *F. tularensis* ssp. *holarctica* strains (both genotypes B.12 and B.FTNF002-00). Clinical signs included porphyrin accumulation around the eyes, nasal discharge, weight loss, weakness, ruffled fur, inactivity, diarrhoea and laboured breathing (Table 1). General linear model analysis showed the effect of the challenge dose on the course of the disease (timing of the most severe symptoms), however, association between the severity of the clinical signs and the challenge dose was not detected. More than 50% of the rats survived the intra-peritoneal challenge by *F. tularensis* ssp. *holarctica* strains in all but one group (genotype B.FTNF002-00, 10^0^ CFU), thus further analysis was based on the comparison of the two main groups differing in the genotype of the infectious agent. Total weight loss was significantly higher (*p* = 0.0348; independent *t*-test) in the group infected with the B.FTNF002-00 genotype. Changes in body weight (%) between days 3 and 13 post infection are shown in Fig. 1, and the percentages of the total weight loss are listed in Table 2. Comparison of the severity of clinical signs (based on clinical scores between days 1–13 post infection) revealed significant differences (*p* = 0.004; Mann-Whitney *U* test) between the two groups. Median values of the clinical scores and changes in weight loss between days 1 and 13 post infection are demonstrated in Fig. 2. The median values of the clinical scores between days 1–13 post infection showed, that rats infected with the B.12 genotype recovered from the disease two days earlier than rats infected with genotype B.FTNF002-00. In the B.FTNF002-00 genotype infected groups 33.3% (6/18) of the animals succumbed to tularemia between days 4–12 post infection. In contrast, only 11.1% (2/18) of the rats died of the disease caused by the B.12 genotype on days 8 and 10 post infection. Post mortem examinations did not reveal differences in the pathological changes associated with the two bacterial strains. At necropsy, the deceased rats were seronegative while all surviving rats showed seroconversion in slide agglutination test on day 21 post infection (Table 2). Macroscopic pathological findings were scarce, an enlarged spleen being occasionally observed in both deceased and euthanized rats. Histological findings in rats that succumbed to the infection consisted of acute multiple necrotic foci in the liver and spleen and immunohistochemistry showed large amounts of antigen in these organs (Fig. 3). Sub-acute interstitial lympho-histiocytic inflammation was also observed in the lung with high or moderate amounts of antigen in rats that died of the infection. Seropositive rats which were sacrificed on day 21 post infection showed distended sinusoids and activation of Kupffer cells associated with multifocal interstitial infiltration by mixed inflammatory cells in the liver and a moderate follicular hyperplasia and hyperaemia in the spleen with no or low amounts of antigen (Table 2).

**Table 2:** Clinical and post mortem data of Fischer 344 rats (6 rats/groups) infected with *Francisella tularensis* ssp. *holarctica*

Strain	Infectious dose	Clinical sign^a^	Total weight loss (%)	Day of death^b^	Seroconversion	Amount of antigen (IHC)^c^
B.12	10^0^ CFU	mild	0	E	+	low
			0	E	+	minimal
			2.5	E	+	none
			3.5	E	+	none
		severe	7.3	E	+	low
			24	10	-	high
	10^1^ CFU	mild	0	E	+	minimal
			0	E	+	low
			3	E	+	minimal
			10.6	E	+	minimal
		moderate	3.6	E	+	minimal
		severe	18.1	8	-	high
	10^2^ CFU	mild	0	E	+	minimal
			3.7	E	+	none
			7	E	+	minimal
			17.6	E	+	minimal
		moderate	4.6	E	+	low
			13.6	E	+	minimal
B.FTNF002-00	10^0^ CFU	mild	0	E	+	none
			3.1	E	+	none
			14.4	E	+	minimal
		severe	5.6	7	-	high
			11.4	8	-	high
			35.5	12	-	high
	10^1^ CFU	mild	5.7	E	+	none
			11.4	E	+	none
		moderate	9	E	+	low
		severe	12.4	7	-	moderate
			16.5	E	+	low
			21.5	9	-	moderate
	10^2^ CFU	mild	4.8	E	+	none
			6.3	E	+	none
		moderate	7.7	E	+	minimal
			9.3	E	+	low
			19.3	E	+	none
		severe	12.3	4	-	high

^a^The most severe clinical sign shown during the experiment

^b^Day of death after injection; E: euthanized on day 21 post infection

^c^During the immunohistochemical examinations the amount of antigen was evaluated in the lung, liver and spleen and was based on the number of affected organs and antigen observations/field of vision at 100× magnification

## Discussion

While clear differences are described in the virulence of the *F. tularensis* ssp. *tularensis* subpopulations, little or no information is available about the virulence of the subpopulations of the widespread *holarctica* subspecies [23]. Interactions among hosts with different susceptibility to tularemia and strains of the two genetic groups of *F. tularensis* ssp. *holarctica* (B.12 and FTNF002-00) were previously compared in vitro, but no difference was detected in the ability of genotypes to evade the hosts’ complement system [24].

The susceptibility of Fischer 344 rats to tularemia was described in previous examinations [17, 19, 20]. Experimental infection of Fischer 344 rats with intra-peritoneal inoculation of 10^1^ CFU of a *F. tularensis* ssp. *holarctica* strain from Sweden (B.12 genotype) induced fatal disease within 10 days [18].

The results of the current study revealed moderate difference in the pathogenic potential of the two strains belonging to the two genetic groups of *F. tularensis* ssp. *holarctica* and support the hypothesis that B.FTNF002-00 genotype is more virulent than the B.12 genotype. Low mortality rates were observed in the infected animals, which might be an effect of a possible attenuation process during the culture of bacteria on artificial media and implies the potential need for the use of higher bacterial loads in experimental infections. However, clinical signs manifested in all rats, and they were in accordance with previously described symptoms in Fischer 344 rats infected subcutaneously with *F. tularensis* ssp. *tularensis* SCHU S4 strain [19]. Interestingly, the challenge dose did not affect the severity of clinical signs, the time and number of deaths, nor the extent of weight loss, similarly to the observations in previous studies on non-human primates and Fischer 344 rats [19, 25]. Analysis of weight loss was used for the determination of susceptibility of Fischer 344 rats to tularemia before [19]. In the current experiment rats infected with B.FTNF002-00 genotype showed significantly higher total weight loss and more prolonged course of the disease compared to rats infected with B.12 genotype (Figs. 1 and 2), suggesting a more severe progression of the illness during infection with the B.FTNF002-00 genotype. The assumed higher virulence of B.FTNF002-00 genotype is consistent with previous observations on the clinical features and pathology of tularemic brown hares associated with the two genotypes. Nevertheless, experimental infection of the brown hare, the host presenting different types of pathological changes, with a higher number of strains belonging to these two genotypes should confirm field observations and enable deeper insight into the assumed difference in virulence between genotypes. Examining the *F. tularensis* ssp. *holarctica* susceptibility of European brown hares originating from distinct geographical areas may also provide important additional information about the ecology of this pathogen.

## Conclusion

The moderate differences in the pathogenic potential of B.12 and B.FTNF002-00 genotype *F. tularensis* ssp. *holarctica* strains, as observed during the experimental infection of Fischer 344 rats support a higher virulence of B.FTNF002-00, which is the dominant genotype in Western European countries. The results are in accordance with previous observations on the pathology of tularemic brown hares infected by the two genotypes.

## Additional file

Additional file 1: Weight data and clinical sign scores of *Francisella tularensis* ssp. *holarctica* infected Fischer 344 rats. The animals were infected with the B.12 or the B.FTNF002-00 genotype (genotype of infectious agent) with either 10^0^, 10^1^ and 10^2^ CFUs of the bacteria suspensions (infectious dose). Weight data (in grams) on the day of infection (initial weight) and between days 1–13 post infection (pi.) with the clinical sign scores (graded as none = 0, mild = 1, moderate = 2 and severe = 3; 3* = the animal succumbed to the infection) are provided for all rats. (XLSX 14 kb)

## Abbreviations

CFU: Colony forming units
HRP: Horse-radish peroxidase
LVS: Live vaccine strain
PBS: Phosphate-buffered saline
SNP: Single-nucleotide polymorphism
